# Cardamonin inhibits colonic neoplasia through modulation of MicroRNA expression

**DOI:** 10.1038/s41598-017-14253-8

**Published:** 2017-10-24

**Authors:** Shirley James, Jayasekharan S. Aparna, Aswathy Mary Paul, Manendra Babu Lankadasari, Sabira Mohammed, Valsalakumari S. Binu, Thankayyan R. Santhoshkumar, Girijadevi Reshmi, Kuzhuvelil B. Harikumar

**Affiliations:** 1 Cancer Research Program, Rajiv Gandhi Centre for Biotechnology, Thiruvananthapuram, Kerala State 695014 India; 2 Computational Biology Group, Cancer Research Program, Rajiv Gandhi Centre for Biotechnology, Bio-Innovation Center, KINFRA Park, Thiruvananthapuram, Kerala State 695585 India; 3 Manipal University, Manipal, Karnataka State 576104 India; 4 Department of Statistics, Manipal University, Manipal, Karnataka State 576104 India

## Abstract

Colorectal cancer is currently the third leading cause of cancer related deaths. There is considerable interest in using dietary intervention strategies to prevent chronic diseases including cancer. Cardamonin is a spice derived nutraceutical and herein, for the first time we evaluated the therapeutic benefits of cardamonin in Azoxymethane (AOM) induced mouse model of colorectal cancer. Mice were divided into 4 groups of which three groups were given six weekly injections of AOM. One group served as untreated control and remaining groups were treated with either vehicle or Cardamonin starting from the same day or 16 weeks after the first AOM injection. Cardamonin treatment inhibited the tumor incidence, tumor multiplicity, Ki-67 and β-catenin positive cells. The activation of NF-kB signaling was also abrogated after cardamonin treatment. To elucidate the mechanism of action a global microRNA profiling of colon samples was performed. Computational analysis revealed that there is a differential expression of miRNAs between these groups. Subsequently, we extend our findings to human colorectal cancer and found that cardamonin inhibited the growth, induces cell cycle arrest and apoptosis in human colorectal cancer cell lines. Taken together, our study provides a better understanding of chemopreventive potential of cardamonin in colorectal cancer.

## Introduction

Colorectal cancer (CRC) is the third most common cancer, and its frequency is increasing^1^. Based on GLOBOCAN2012 estimates, it is the third most common cancer in men and stands second in women and approximately 25% of the patients present with metastatic disease at the time of diagnosis^2^. MicroRNAs (miRNAs) belong to the class of an endogenous small noncoding RNA family and control post-transcriptional gene regulation^3,4^. The association between miRNA and CRC has been reported by several studies which shows oncogenic and tumor suppressive role of miRNAs in CRC^5–7^.

Compelling evidence drawn from multiple epidemiological, clinical and laboratory studies established a critical link between CRC and nutritional components^8^. Dietary compounds can act as chemopreventive agents in CRC, and many such compounds regulate the functions of miRNAs there by modulating the pathways regulated by these miRNAs in several diseases including cancer^9–11^. Cardamonin (2′,4′-dihydroxy-6′-methoxychalcone) belonging to the chalcone class is one such compound initially isolated from members of Zingiberaceae family^12^. Cardamonin inhibited the growth of several cancer cell types including breast cancer^13^, glioblastoma^14^, ovarian^15^, prostate^16^, and lung^17^. This chalcone also sensitized tumor cells to TRAIL induced apoptosis^18^ and inhibited the metastasis of Lewis lung carcinoma cells^19^.

Our major focus is to develop effective strategies for the chemoprevention of colorectal cancer with the aid of dietary and nutritional components. In this study, we for the first time examined the *in vivo* efficacy of cardamonin in an Azoxymethane (AOM) induced CRC model, which shows similarities with spontaneously arising colorectal carcinoma in humans and *in vitro* efficacy using human colorectal cancer cell lines. We found that cardamonin exhibited significant anti-cancer activity in this model. To seek the mechanism of action of cardamonin *in vivo* we focused on the contribution of miRNAs in the process and our findings indicate that modulation of miRNA expression as one of the mechanisms through which cardamonin inhibits colon cancer.

## Results

### Cardamonin inhibited AOM induced colorectal cancer (CRC)

**Figure 1:**
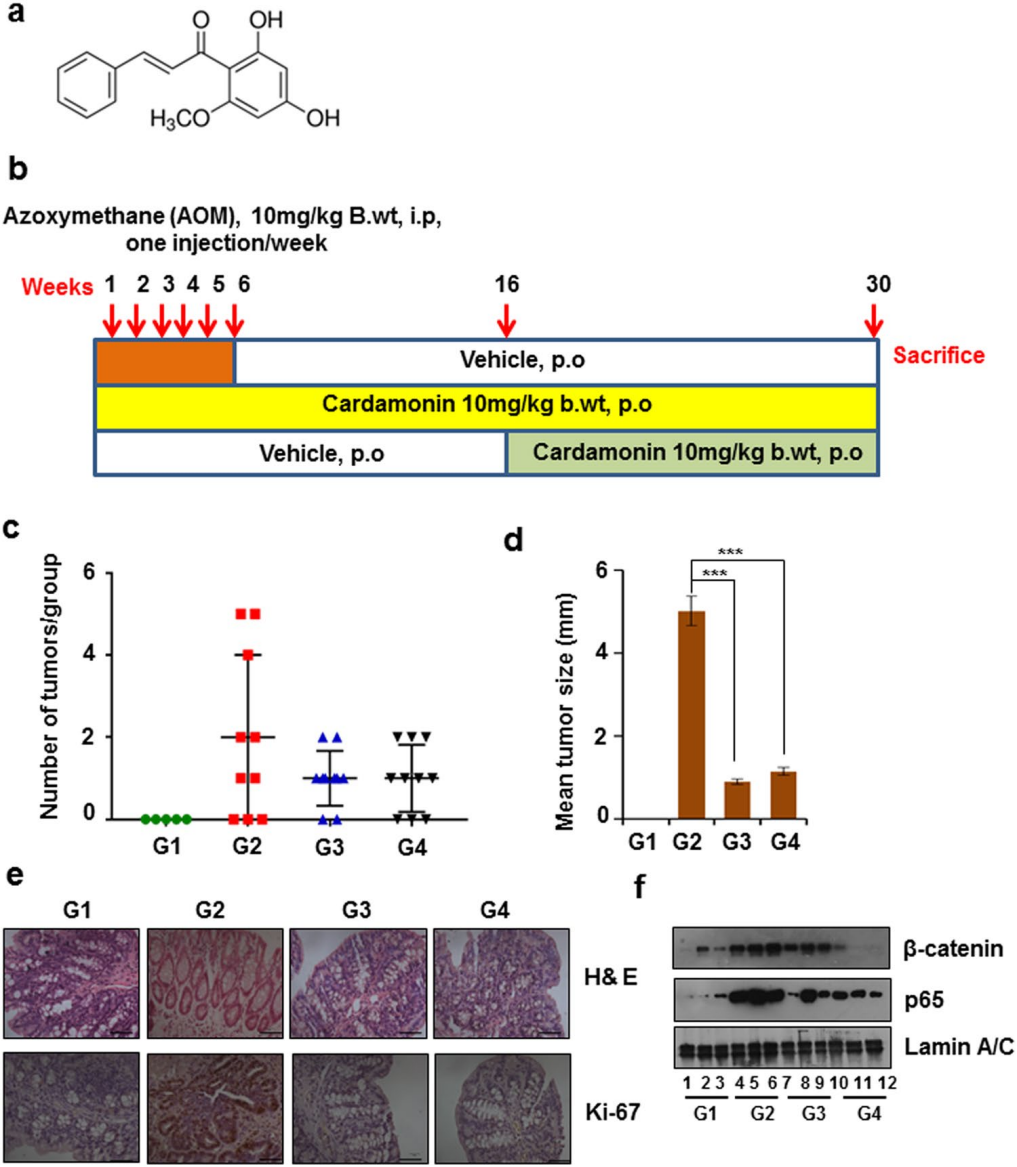
Cardamonin inhibited AOM induced colorectal cancer: (**a**) Chemical structure of cardamonin; **(b)** Schematic representation of experimental design; **(c)** Number of tumors in colon/ group; **(d)** Mean tumor size between groups expressed as mean ± SEM. The statistical analysis was done using one way ANOVA followed by Tukey HSD posthoc test; ***p < 0.001; **(e)** Tissue sections were prepared from formalin fixed paraffin embedded samples. Hematoxylin-eosin staining (upper panel) and Ki-67 index (lower panel) of colonic tissue samples, representative images, 40X magnification; **(f)** Nuclear fractions were prepared from all the 4 groups (n = 3) and western blot analysis was performed for β-catenin (upper panel) and p65 (lower panel). Lamin A/C was used as the loading control.

Azoxymethane-induced colorectal cancer is a well-known pre-clinical model for cancer chemopreventive studies^20,21^. We adopted this model to test the efficacy of cardamonin (Fig. 1a and b) in colorectal cancer initiation and progression. Development of CRC in AOM model closely mirrors the pattern of CRC incidence observed in humans^20^. The colonic epithelial cells undergo sequential pathological changes after treatment with AOM from aberrant crypt foci (ACF), to adenoma and malignant adenocarcinoma. The ACF formation is observed after 14–16 week after initial AOM administration. Therefore we decided to include two treatment groups for cardamonin. Treatment in one group started simultaneously (G3, chemopreventive model) and another group (G4, developed model) started from 16 week after AOM treatment. Mice in all the four groups were regularly monitored for their body weights and food intake and did not observe any considerable difference between the groups (data not shown). There was unexpected death of two animals from group 2 in the second week of the study. We could not determine the cause of the death after morphological and pathological investigations and considered this as non-specific death and not due to experimental conditions. In cardamonin treated groups we also checked for any cardamonin associated adverse toxicity. Necropsy was performed after sacrifice and we did not observe any gross morphological or pathological alterations in major organs (liver, kidney, lung and spleen). The percentage of tumor incidence is shown in Supplementary Table 1. Two animals in group 2 and three animals in group 4 did not develop any tumors. Figure 1c depicted the number of tumors in each mouse. We also calculated the tumor multiplicity in both the treatment groups and found that there was a 40% decrease (p < 0.05) in tumor multiplicity. The tumor size distribution (Fig. 1d) provides a better scenario regarding the efficacy of cardamonin. Compared to AOM alone treated groups the mean tumor size were significantly reduced in cardamonin treated groups (p < 0.001). The tumors that arose in the treatment groups were smaller in size. Mice from group 1 (untreated group) did not show any tumor incidence. Most importantly, cardamonin treatment after the development of tumors produced significant anti-cancer effect in comparison to simultaneous treatment group. Moreover, the percentage of tumor incidence was 70% in group 4 as compared to 80% in group 3 (Supplementary Table 1). Furthermore, we did not observe any apparent toxicity of cardamonin at the given doses over a period of 30 weeks (treatment duration). Together, our results show the preventive effect of cardamonin in CRC.

### Cardamonin inhibits cell proliferation and promotes apoptosis

A histological examination of tissue samples using hematoxylin-eosin staining was performed. The colonic sections of group 1 showed normal histological architecture with regular rounded crypts. In group 2 severe inflammation associated with dysplasia was observed. The crypts showed hyperplasia along with increased thickening of the mucosal layer. However these changes were greatly attenuated in cardamonin treated groups (Fig. 1e upper panel). We performed an immunohistochemical analysis of tissue samples for Ki-67. The tissue sections of group 2 expressed higher levels of Ki-67 compared to group 1 and in cardamonin treated groups the Ki-67 index was substantially reduced indicating that administration of this chalcone inhibited cell proliferation (Fig. 1e lower panel). β-catenin, is a key molecule in the Wnt/β-catenin pathway plays a major role in cellular proliferation and the role of this protein in AOM models of CRC is already been established^22^. We performed a western blot analysis for β-catenin and observed that nuclear translocation of β-catenin was more evident in group 2 further confirming that cardamonin reduced the cell proliferation (Fig. 1f).

### Cardamonin inhibited the NF-κB signaling and p65 translocation

NF-κB regulates various genes involved in inflammation and tumorigenesis^23^. Western blot analysis of nuclear fractions with an antibody specific for p65 demonstrated that more nuclear translocation of p65 was observed in group 2 (the tumor group). The lower level of NF-κB translocation was observed in both cardamonin treated groups indicating that cardamonin was effective in preventing NF-kB activation (Fig. 1f).

### MicroRNA expression was altered after cardamonin treatment

**Figure 2:**
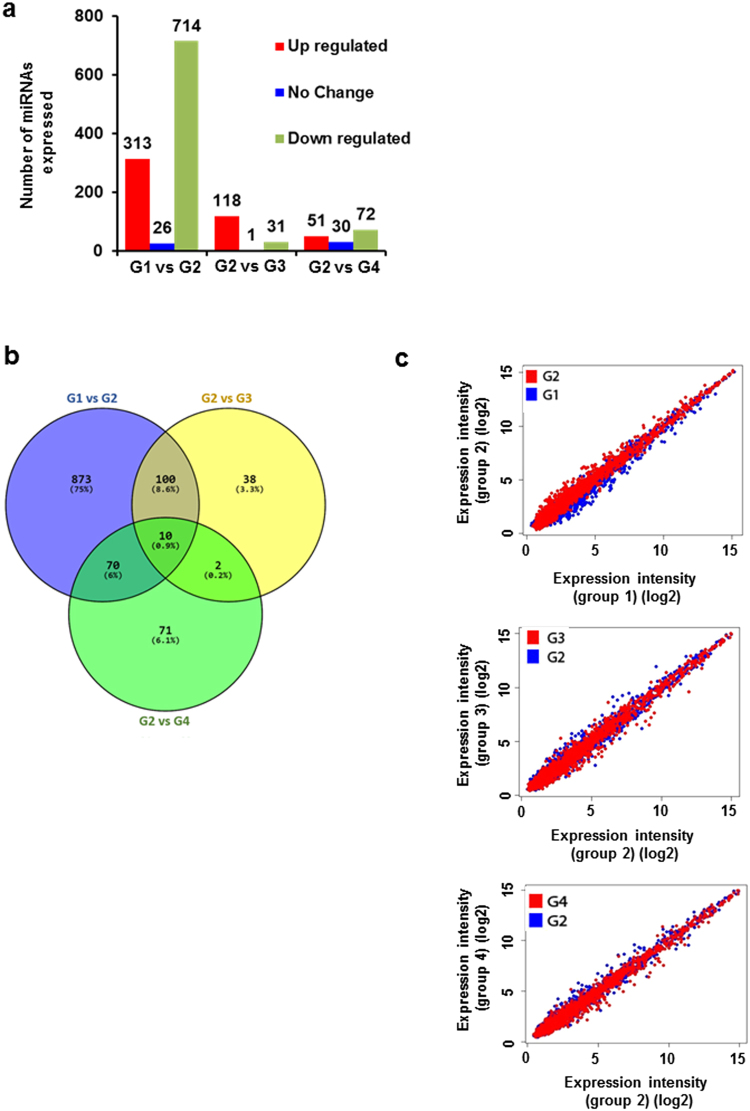
MicroRNA expression were altered after AOM and cardamonin treatment: Group 1 (normal control) was compared with Group 2 (vehicle control) and group 2 was further compared with group 3 and 4 (Cardamonin treated groups) **(a)** The number of miRNA expressed between the groups; **(b)** Venn diagram comparing the expression between groups; **(c)** scatter plot showing miRNA expression intensity values (log scale).

**Figure 3:**
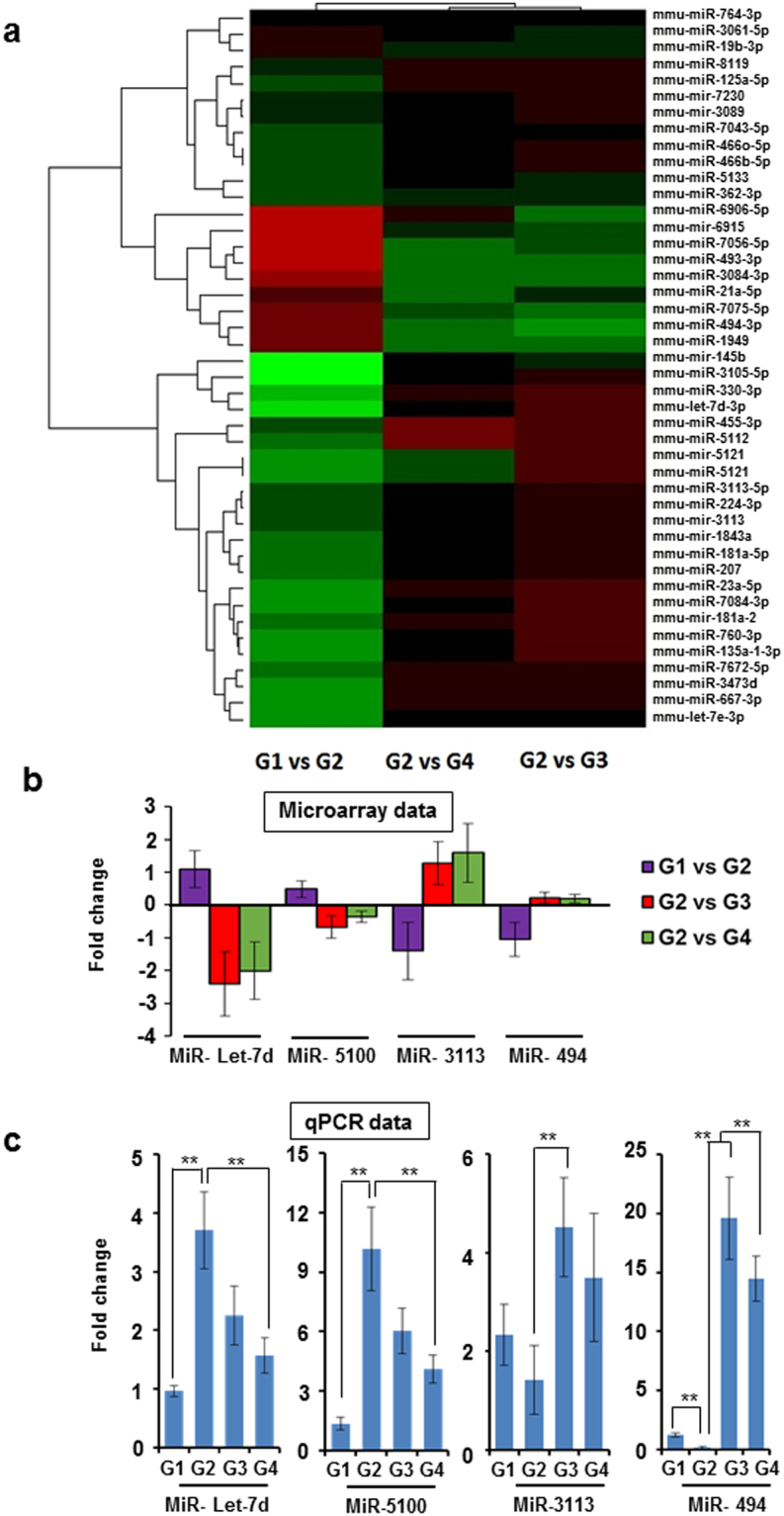
MicroRNA expression were altered in colorectal cancer: (**a**) Heat map plot showing the differentially expressed miRNAs with highest standard deviation (p ≤ 0.10); **(b)** Log fold change values obtained after microarray analysis for selected miRNAs between groups; **(c)** The validation data of selected miRNAs in all the four groups. The total RNA was isolated from each sample and expression of each miRNA was analyzed using specific Taqman probe. The sno202 was served as internal control. The data is expressed in fold values and expressed mean ± SD, statistical analysis was done using student’s t test; **p < 0.01.

The possible mechanism of action of cardamonin in preventing the progression of colorectal cancer in mice was investigated. We seek to understand the role played by miRNAs and how the treatment of cardamonin affected the global miRNA expression in CRC. To obtain the comprehensive profiles of different miRNAs in CRC, we did a global miRNA profiling. The number of miRNAs observed between the groups is shown in Fig. 2a and as Venn diagram in Fig. 2b. We observed that the treatment with AOM significantly altered the miRNA expression. Initially we compared the expression level between group 1 and 2 to see the altered miRNA profiles in AOM alone group as compared to untreated group. Further, group 2 was compared with remaining groups to see the effect of cardamonin in miRNA expression. The scatter plot (Fig. 2c) indicates that expressions of miRNAs were increased in group 2 (tumor control) as compared to group 1 (untreated group). We set a cutoff value of 1.5 which will eliminate background noise and only a few genes will be left for the further analysis. Supplementary Table 2 shows the statistically most significant (p ≤ 0.05) miRNA in each group. Figure 3a shows the heat map of significant miRNAs with highest standard deviation (p ≤ 0.05). Eight miRNAs which were downregulated in group 2 were significantly upregulated after cardamonin treatment. Similarly, three miRNAs which were upregulated in group 2 were reduced in treatment groups.

### Validation of selected microRNA samples

For validation, we selected 4 miRNAs from Supplementary Table 2. The average expression value (log transformed) of each miRNA based on microarray data is shown in Fig. 3b. We found that 3 miRNAs were highly upregulated in group 2 and out of which the role of miR-let7 in colorectal cancer is already known. However there were no reports about miR-5100 and 7066-3p in colorectal cancer. We decided to select miR-5100 because of reported effects in promoting tumor growth in lung and pancreatic cancer cells. MiR-let7 and miR-5100 were upregulated in group 2 and expression was found to be decreased after cardamonin treatment. A statistically significant difference was observed in group 4 (where the treatment started after the development of tumor; p < 0.01) for both miRNAs. There were 8 miRNA down regulated in group 2 after AOM treatment. Except miR-3113 the role of other miRNAs in cancer is already established. The miR494 was recently included in the panel of microRNAs recommended for the detection of early relapse in postoperative colorectal cancer patients. Therefore we selected MiR-494 and miR-3113 for validation. The miR494 expression was restored in both cardamonin treated groups (p < 0.01). However a statistically significant upregulation of miR-3113 was observed in group 3 only (Fig. 3c).

### Predicted network of miRNA- gene interaction

**Figure 4:**
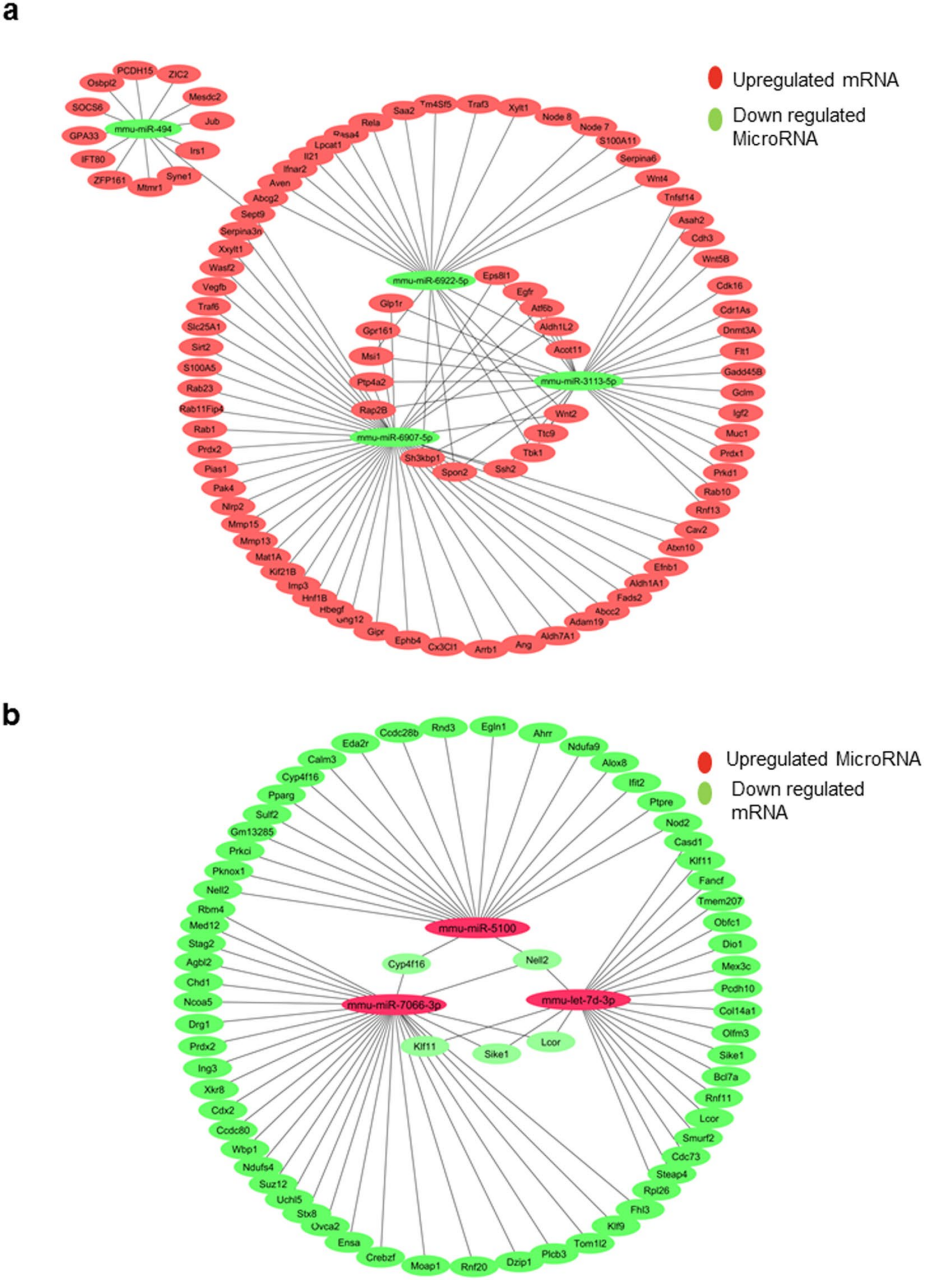
The microRNA-gene interaction network in CRC: The network was visualized using cytoscape for the table of miRNA and mRNA target interaction and displayed as circular architecture: (**a)** with top 4 upregulated miRNAS (green in color) with p ≤ 0.05 and targeting downregulated mRNA red in color) (**b**) with top 3 downregulated miRNAs (red in color) with p ≤ 0.05 and targeting upregulated mRNA (green in color).

Further, we decided to construct a gene interaction network with selected miRNAs and their putative targets. We searched four databases to identify the targets which have relevance to cancer. The prediction is based on the sequence complementarity in 5′ and 3′ UTRs between miRNA and target genes (Supplementary Table 3). We selected targets of following miRNAs to construct the interaction network viz. mmu-miR-6922-5p, mmu-miR-6907-5p, mmu-miR-3113-5p, mmu-mir-494 (down regulated in group 2), mmu-miR-5100, mmu-miR-7066-3p, and mmu-let-7d-3p (upregulated in group 2). The network of up regulated and down regulated miRNAs are shown in Fig. 4a and b. The selection of the targets was done from Targetscan with a cutoff of less than −0.36. These genes were manually sorted based on their roles in cancer and the network was made using Cytoscape^24^.

**Figure 5:**
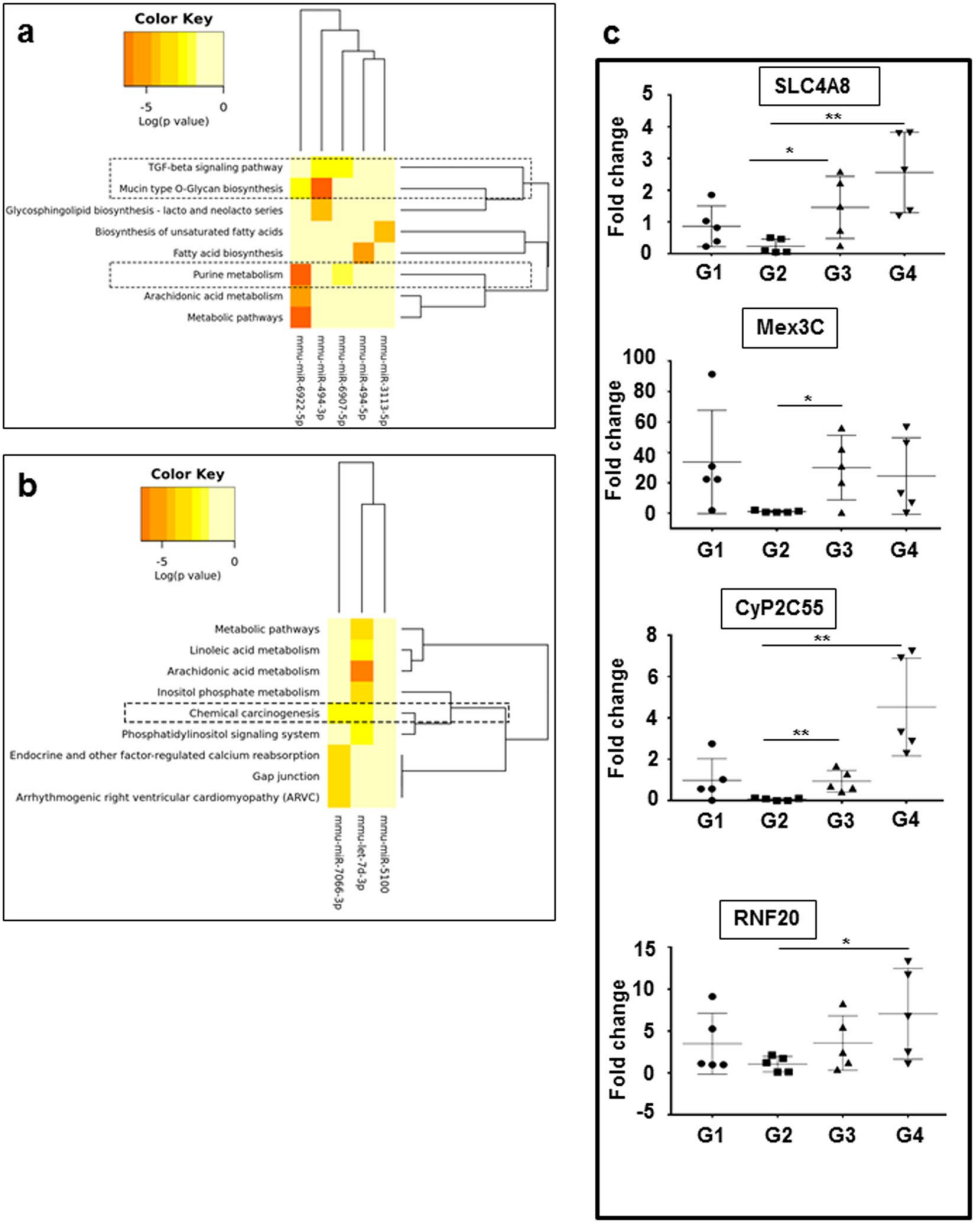
The correlation between microRNA expression and enriched functional pathways: Dendrogram plot showing down regulated miRNAs **(a)** and upregulated **(b)** versus significantly enriched functional pathways. In both figures darker colors represent statistical significance as indicated in the color key. Pathways within dotted box are the functionally relevant pathway clusters regulated by more than one miRNA. **(c)** The validation data of selected genes using RT-PCR. Statistical analysis was done using student’s t test; *p < 0.05, **p < 0.01.

Next we used DIANA-miRPath v3.0 to identify the link between selected miRNAs and various signaling pathways^25^. We preferred cancer associated biological functions and related signaling cascades. A dendrogram, that shows the relationship between the miRNAs, was constructed and was implemented using unsupervised hierarchical clustering. The intensity of the color signifies log p-value (Fig. 5a,b). The dotted box represents those pathways which are regulated by more than one miRNA.

To validate the above findings, a set of genes whose expression is regulated by the selected miRNAs was identified. MiRNAs which are common among at least two databases were selected for further analysis. For identification 3′ UTR sequence of target mRNA was taken from the UCSC genome browser^26^ and miRNA sequence from the miRBase database. MicroRNA is hybridized to the target mRNA in an optimal way yielding the minimum free energy (MFE) using the RNAhybrid web server (https://bibiserv2.cebitec.uni-bielefeld.de/rnahybrid). Integrating the MFE calculations into prediction methods will help us to identify energetically stable base pairing between microRNA and target sequence. We selected the energy cutoff as below −17.0 Kcal/mol and selected 11 targets genes (Supplementary Table 3). We selected 4 genes for validation, which were SLC4A8, RNF 20, MEX3C and Cyp2C55 (Supplementary Table 5).

### Validation of selected gene targets

A quantitative PCR approach was used for analyzing the selected gene targets in all the four groups. SLC4A8 is regulated by mmu-miR-6939-5p and expression of this miRNA was not statistically significant among the groups. We observed a similar trend among all the four groups (Fig. 5c). Mmu-let-7d-3p regulates the expression of both Mex3c and CyP2C55. Both genes were down regulated in group 2 and the expression was increased after cardamonin treatment. RNF20 is a potential target gene whose expression is controlled by mmu-miR-7066-3p. RNF20 is reported to be lost during carcinogenesis. However, we noticed that RNF20 expression was restored after cardamonin treatment (Fig. 5c)

### Cardamonin alters redox signaling through modulation of miRNA

**Figure 6:**
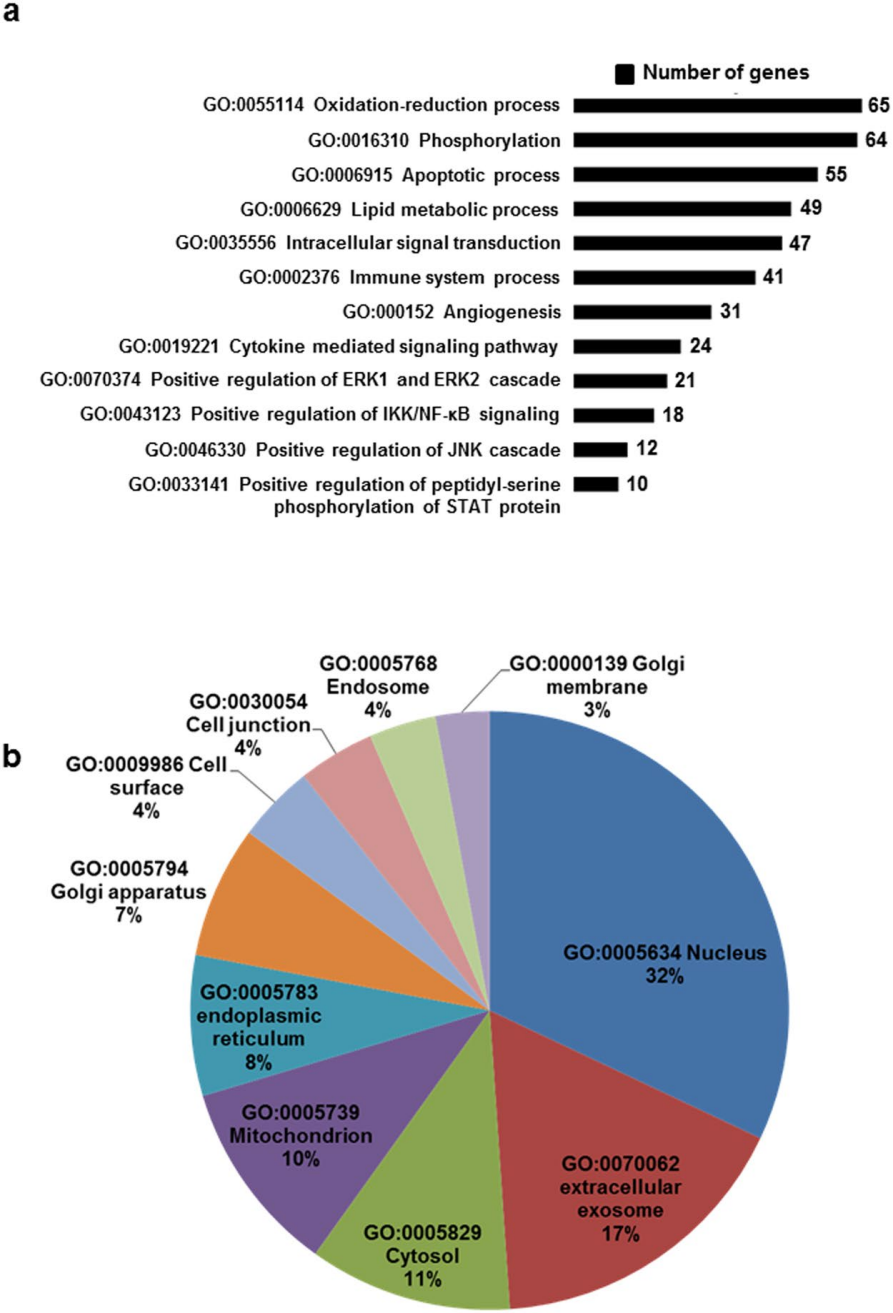
Functional annotation of genes using the Database for Annotation, Visualization and Integrated Discovery (DAVID) enrichment analysis: (**a**) The nodes represent the GO_BP terms in the enrichment analysis. The size of a node represents the number of genes associated with each pathway (**b**) Pie chart showing the expression of organelle specific expression of each gene.

Next we sought to know the major biological processes, which were altered. To address this we performed gene ontology enrichment analysis using the Database for Annotation, Visualization and Integrated Discovery (DAVID), Version 6.7 (http://david.abcc.ncifcrf.gov/)^27^. We took all the miRNA gene targets from TargetScan with a cut-off of less than −0.36 and the selected targets were functionally annotated. Majority of the altered proteins belong to oxidation-reduction processes, apoptosis, lipid metabolism, angiogenesis and NF-κB pathways. The most strongly represented pathways are represented in Fig. 6a. Next we did fold enrichment in cellular components to check which proteins of particular cellular localization are involved. Majority of proteins are nuclear localized with (32%), exosomes (17%), cytosol (11%), mitochondria (10%), endoplasmic reticulum (8%), Golgi apparatus (7%) and in few minor organelles (Fig. 6b). These two data strongly revealed the alteration in mitochondrial and ER proteins, which is a prime source of redox reactions which may lead to oxidative stress, free radical generation and peroxidation reactions and would be further elucidated in cell culture based system.

### TCGA data analysis for miRNAs in human CRC

We also searched the TCGA database to see the relevance of our miRNA data to human colorectal cancer^28^. Differential expression analysis identified 460 miRNAs with an FDR cut-off less than 0.01.We then compared the list with miRNA identified in mouse CRC and found that hsa-miR-let-7d is found to be expressed in human CRC. However, an inverse trend in human CRC samples was observed in comparison with mouse tumor samples. Let-7d expression was down regulated in human tumor samples (Supplementary Figure 1).

### Cardamonin inhibited the proliferation of human colorectal cancer cell lines ***in vitro***

**Figure 7:**
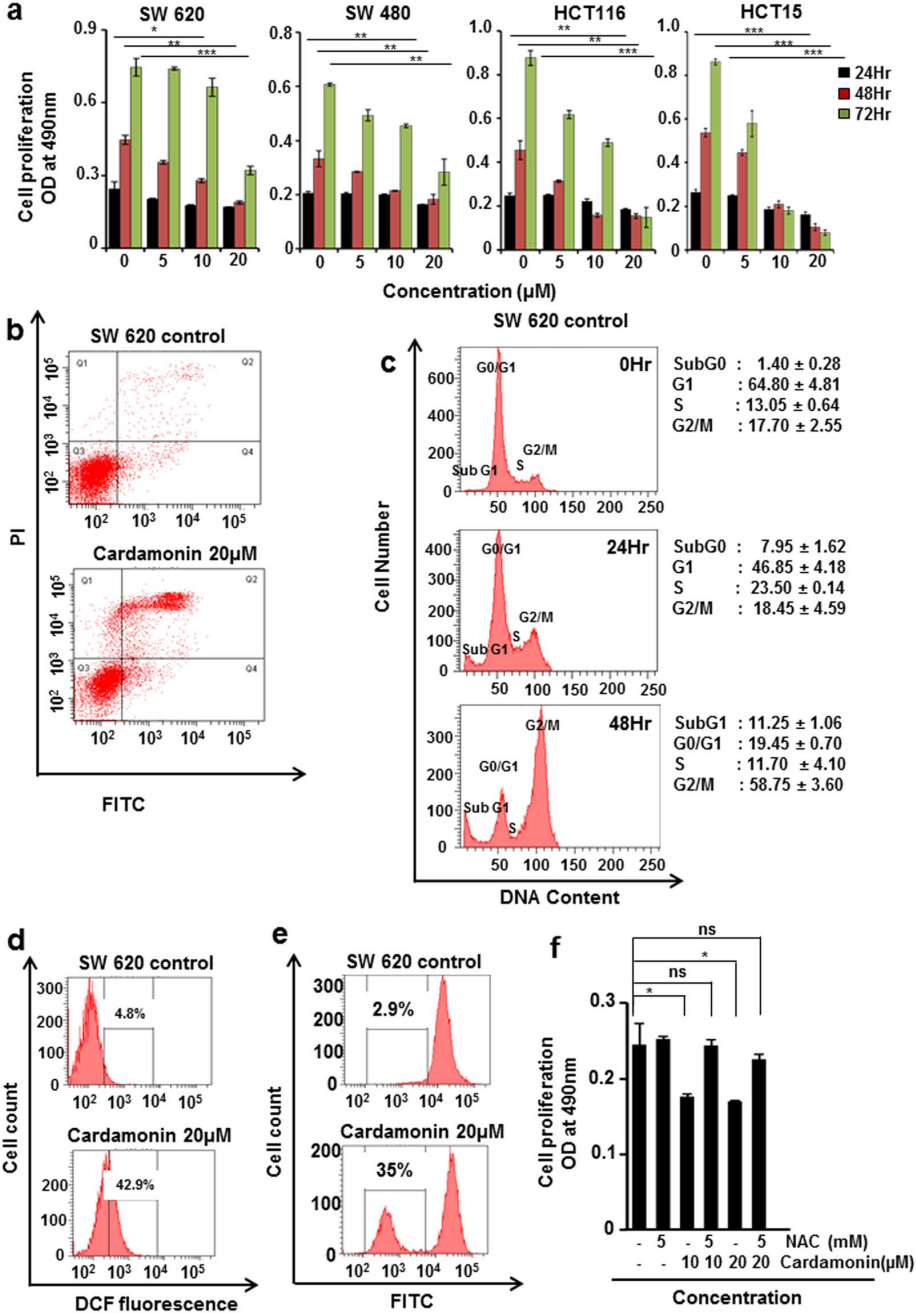
Cardamonin inhibited the growth CRC cell lines *in vitro* **(a)** SW620, HCT15, SW480 and HCT116 cells were treated with the indicated concentrations of cardamonin for 24, 48 and 72 hours. Cell viability was accessed using MTT assay, student’s t test *p < 0.05, **p < 0.01, ***p < 0.001 compared to control, OD: optical density. **(b–f)** SW620 cells were treated with 20 µM of cardamonin for different time points. **(b)** After 24 hours cells were stained with Annexin V5-FITC and PI and then analyzed by FACS **(c)** After 24 and 48 hours cells were stained with PI and cell cycle analysis done by FACS **(d)**; Cells were stained with DCFDA and ROS generation was analyzed by FACS **(e)** after 24hrs the loss in mitochondrial membrane potential was quantified using DiCO6(3) staining. The experiments were repeated at least 3 times and data was expressed as mean ± S.D **(f)** Cells were pretreated with or without NAC (5 mM) for 2hr followed by treatment with or without cardamonin at indicated doses for 24 hr. Cell viability was accessed using MTT assay, student’s t test; *p < 0.05, ns- not significant p > 0.05 compared to control.

Since cardamonin shows promising efficacy in mouse model of colon cancer, we decided to extend our findings to human colorectal cancer. We first investigated whether cardamonin can inhibit the growth of human CRC cell lines and found that this chalcone inhibited the cell proliferation of all the four cell lines tested in a dose dependent manner (Fig. 7a). We selected SW620, a metastatic cell line, for further *in vitro* studies

### Cardamonin induces apoptosis in CRC cells ***in vitro***

To investigate the pro-apoptotic effect of cardamonin, we did Annexin staining of SW620 cells after treatment. Cardamonin treatment increased the apoptotic population from 6.9% (in control group) to 31.3% after treatment (Fig. 7b).

### Cardamonin caused cell cycle arrest

We found that cardamonin induces apoptosis in CRC cell lines. We further investigated whether the inhibition of cell proliferation caused by cardamonin is through induction of apoptosis or cell cycle arrest or simultaneously activating both the processes. We treated SW620 cells with cardamonin for 24 and 48 hours and cells were stained with PI and cell cycle analysis was carried out. We found that treatment of cardamonin induces cell cycle arrest mostly in the S phase of cell cycle (Fig. 7c). These studies confirmed that cardamonin activates both apoptosis and induces cell cycle arrest to inhibit the cell proliferation.

### Cardamonin increased the generation of ROS production

To investigate whether cardamonin causes an increase in ROS generation to induce apoptosis, we treated SW620 cells with cardamonin and stained with DCFDA followed by analysis of cell population using flow cytometry. We found that the DCF fluorescence increased from 4.8% (in control) to 42.9% after cardamonin treatment (Fig. 7d), indicating that Cardamonin induces apoptosis in CRC cell line through generation of ROS.

### Cardamonin treatment increase the mitochondrial depolarization

We next did an assay for mitochondrial membrane potential (MMP) and found that both treatment with cardamonin promoted mitochondrial depolarization (2.9 to 35%) indicating that in cardamonin induced apoptosis there is an involvement of mitochondria (Fig. 7e).

### Cardamonin induced ROS and cell death is attenuated in presence of ROS scavenger

To confirm the involvement of ROS in cardamonin induced apoptosis we did the cell proliferation in presence and absence of N-acetyl-L-cysteine^29^ (NAC, a ROS scavenger) in SW620 cells. Data from MTT assay showed that NAC alone did not induce any cell death however pretreatment of cells with NAC rescued the cells from the cytotoxic effects of cardamonin (Fig. 7f).

**Figure 8:**
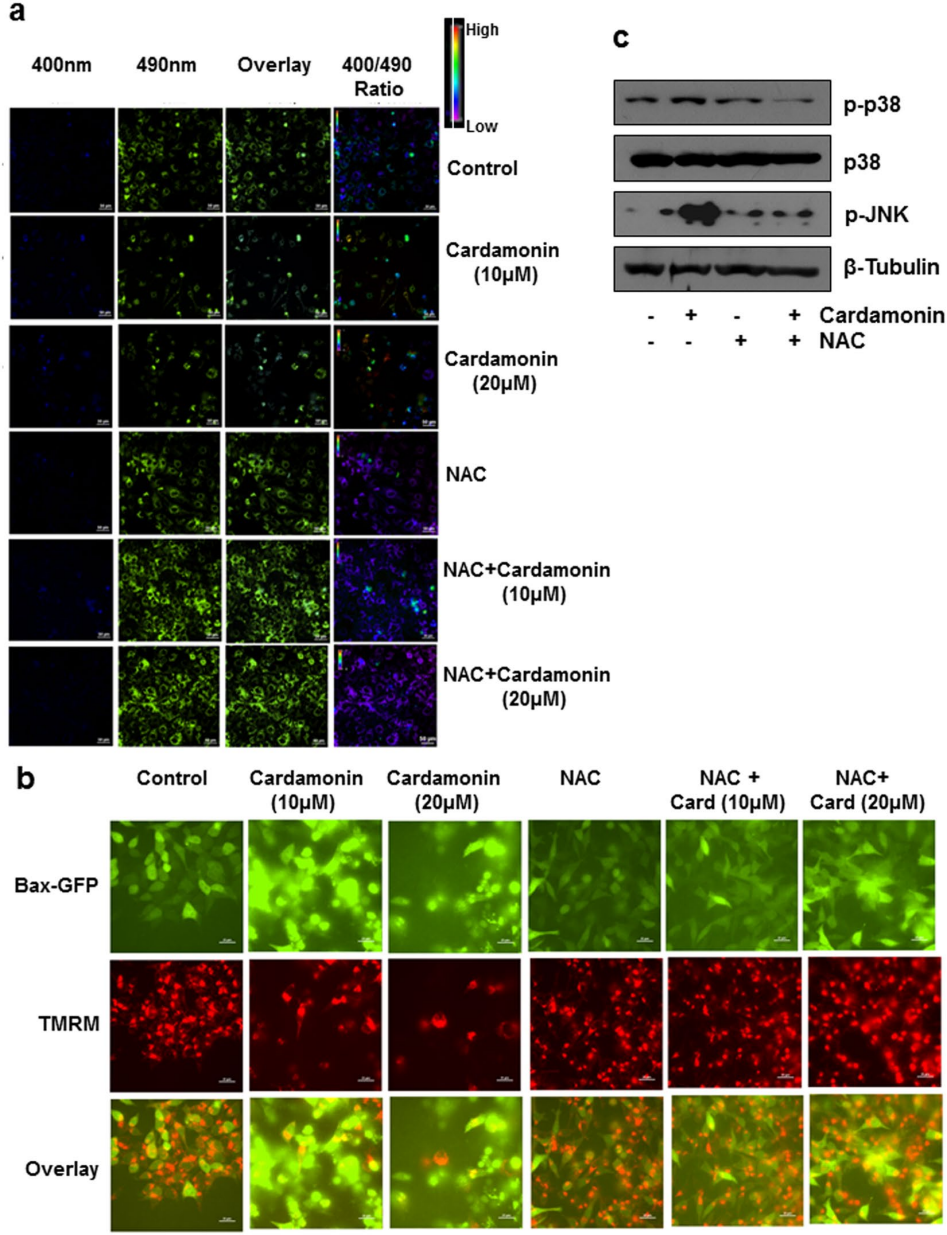
Cardamonin induced apoptosis in a ROS dependent manner (**a)**: HCT116 cells stably expressing a ratio metric redox sensing probe Mito RO-GFP was pretreated with NAC (5 mM) for 2 hr in presence and absence of Cardamonin. The ratio of emission after excitation at 400 nm to 490 nm (400/490) was calculated. Representative images were shown in X; **(b)** HCT116 Bax^−/−^ stably expressing EGFP were pretreated with NAC (5 mM) for 2 hr in presence and absence of Cardamonin for 48 hr and cells were stained with 100 nM of TMRM. Loss of mitochondrial potential is reflected as loss of red fluorescence and Bax punctae reflects its mitochondrial translocation after activation.

Further to prove the essential involvement of oxidation injury and mitochondrial damage, we studied the ROS level using cells stably expressing a ratio metric redox sensing probe Mito RO-GFP, targeted to the mitochondria^30,31^. The biosensor is a GFP variant in which two surface-exposed cysteines are added at positions 147 and 204, sensing the environmental redox status and undergo reversible oxidation- reduction resulting in spectral changes. This protein exhibits dual excitation at ~400 nm and ~490 nm and single emission peak at ~510 nm. The ratio of emission after excitation at 400 nm to 490 nm (400/490) indicates the redox status. The ratio increases when the probe is oxidized and as it is reduced the ratio decreases. There is significant increase in the ratio after treating the cells with cardamonin in a dose dependent manner (Fig. 8a) indicating the generation of ROS. When the cells were pre-treated with NAC before exposing to cardamonin the 400/490 ratio was decreased in both concentrations of cardamonin. Collectively these experiments further substantiate the observation that apoptosis induced by cardamonin is mediated through ROS generation at mitchondria.

### Cardamonin induced the translocation of Bax and concomitant decrease of MMP in colorectal cancer cells

Bcl-2 family contains both pro-and anti-apoptotic factors and there is a complex interplay between these members, which regulate the response to tumor cells to anticancer drugs. Bax is a pro-apoptotic member and colorectal cancer deficient of Bax are resistant to apoptosis and translocation of bax to mitochondria is an important event during apoptosis^32^. We selected HCT116 Bax^−/−^ cells over expressed with Bax-GFP. In untreated cells Bax was localized predominantly in the cytoplasm and staining of the cells with TMRM showed intact mitochondria (Fig. 8b). When cells were exposed to cardamonin there is dose dependent increase in Bax translocation to mitochondria as indicated by dotted puncta with concomitant loss of MMP as showed by loss of TMRM fluorescence. Interestingly both events were significantly inhibited in presence of NAC where cardamonin was unable to induce Bax translocation. This confirms that redox alteration induced by cardamonin is the contributing factor for MMP loss and Bax translocation in colorectal cancer cell lines (Fig. 8b).

### Activation of MAP kinase pathway involved in cardamonin induced apoptosis is decreased in presence of NAC

The mitogen-activated protein (MAP) kinase superfamily controls many biological processes in the cells. Several agents have been shown to activate both c-Jun N-terminal kinases (JNKs) and stress-activated protein kinases (p38 family) MAP kinases thereby leading to cell death in ROS dependent manner^33,34^. When the cells were exposed to cardamonin we could observe significant phosphorylation of both JNK and p38. This observed effect was almost abolished in presence of NAC (Fig. 8c). Interestingly NAC alone did not activate both MAPKs. The results obtained from DAVID analysis and *in vitro* studies in colorectal cancer cell lines further confirm that cardamonin induced cell death is mediated through increased generation of ROS and altering redox status of the cells.

## Discussion

The present study is the first report that provides the scientific evidence for the protective effect of cardamonin on Azoxymethane induced colorectal cancer, a well-established mouse model of CRC. Both the treatment modalities of cardamonin were found to significantly prevent the tumorigenesis. The large sized tumors were mostly absent in cardamonin treatment groups. The analysis of the differential expression of miRNAs after the treatment of AOM and cardamonin showed significant alteration in the global miRNA expression profiles. To the best of our knowledge, this is the first study which examines the miRNA profiles in AOM induced CRC model. However there were few reports where miRNA expression analysis has been done in AOM/DSS model of cancer^35–37^. A selection and validation of miRNAs obtained from the microarray data was carried out. We observed that in AOM alone treated groups there was an upregulation of miR-5100 which was decreased in cardamonin treated groups. Increased level of miR-5100 is reported in lung cancer where exogenous expression of the miRNA increased proliferation and colony formation^38^. Mir-let-7 family is generally considered as a putative tumor-suppressive class of miRNAs^39^. However, we could not observe a tumor suppressive function of let-7d in our CRC model. Instead, the upregulated expression in vehicle control group was decreased after cardamonin administration. Josse *et al*.^36^ also reported similar observation in AOM/DSS model of colon cancer where let-7 was upregulated. MiR-494 is reported to be downregulated in other tumor types^40,41^ which is in agreement with findings from the current study. MiR-3113 is found to be a novel miRNA and we found a reduced expression in tumor samples indicating a putative tumor suppressive role. The levels were significantly upregulated in both the cardamonin treatment groups. Ours will be the first report describing the role of miR-3113 in CRC.

We selected a set of 4 genes for validation based on computational analysis. Both RNF20 and Mex3c are considered as tumor suppressive genes in cancer. Notably reduced expression of RNF20 is being reported in many cancer types^42,43^. Moreover mice with reduced expression of RNF20 were predisposed to chronic colonic inflammation and associated colorectal cancer^44^. In our study, we observed that the expression of RNF20 was restored after cardamonin administration. Interestingly in group 4 where treatment was started after the development of tumor showed statistically significant upregulation of RNF20. Mex3c is identified as cancer chromosomal instability (CIN) suppressor gene in CRC and reported to be downregulated in cancer^45^. We observed a significant down regulation of this gene in vehicle control groups and both treatment modalities significantly restored the expression of Mex3 C. Further both RNF20 and MeX3c regulate replication stress and chromosomal stability^43,45^. CyP2C55 is a member of cytochrome P450 family and the product of this enzyme 19-hydroxyeicosatetraenoic acid (19-HETE) shown to have significant anti-inflammatory effects^46^. Further, a microarray analysis of AOM/DSS model of colon cancer reported that CyPC255 expression was significantly down regulated in mouse colons^37^. Our study indicated that cardamonin upregulated the expression of this gene which is implicated as a therapeutic target in CRC. In sum, our validation data confirmed that cardamonin acted as an activator of tumor suppressor genes and restored their expression in mouse model of colorectal cancer.

We further extended our finding to human colorectal cancer and found that cardamonin inhibited the growth of a panel of 4 cell lines tested. Subsequent studies confirmed that induction of apoptosis, cell cycle arrest, ROS generation, downregulation of MAPK signaling, induction of Bax translocation and loss of mitochondrial integrity were the mechanisms behind the anti-proliferative effect of cardamonin in human CRC cell lines. Several known chemotherapeutic drugs were known to generate mitochondrial driven ROS which induce apoptosis through activation of JNK signaling and a defect in activation confers tumor resistance. Here we observed that cardamonin treatment resulted in the activation of both p38 and JNK and in presence of ROS scavenger the activation was attenuated. Park *et al*. also reported that this chalcone suppressed the β-catenin dependent gene expression in human CRC cell lines^47^. There is a pursuit for the development of small molecules targeting β-catenin signaling pathway and hit-to-lead optimizations is currently an active area of research^48^.

Cardamonin is known to inhibit various signaling pathways which play a major role in the process of inflammation and cancer. This natural product inhibited the phosphorylation and translocation of both STAT3^16^ and NF-κB^49^. Cardamonin also inhibits angiogenesis through downregulation of miR-21 expression^50^. Recently the preclinical pharmacokinetics and ADME characterization of cardamonin in mice was reported^51^. The intestinal permeability study showed that cardamonin is highly permeable with an effective permeability value in ileum is (P_eff_) 3 × 10^−4^ which is highly significant. This study also reported that within 30 minutes of oral dosing, cardamonin is distributed to various tissues. Most importantly this pharmacokinetic study did not report any adverse toxicity of cardamonin^51^.

Currently, there is considerable interest in using dietary intervention strategies to prevent or slow down or reverse the incidence of diseases including cancer. Dietary patterns are linked to CRC incidence and lower rates of CRC occurrence is reported from countries having regular use of spices in their diet. Therefore, our findings and other reported biological activities of cardamonin suggest that it is a promising compound with the potential to be developed as a future chemopreventive agent against colorectal cancer.

## Materials and Methods

### Materials

Cardamonin (>98%) was obtained from Shanghai Standard Biotech Co., Ltd. China. Azoxymethane was purchased from Sigma-Aldrich (St.Louis, MO, USA). All other reagents used in the study were of analytical grade.

### Cell lines

Human colorectal cancer lines (HCT15, HCT116, SW480, and SW620) were obtained from American Type Culture Collection (ATCC, Manassas, VA) and was authenticated and characterized by the supplier. HCT116 Bax^−/−^ was a kind gift from Dr. Dr. Bert Vogelstein. Upon receiving, cells were expanded and frozen stocks were made in liquid nitrogen. During experiment cells were never used for more than 2 months after revival. Cells were cultured in appropriate media in the presence of fetal bovine serum and antibiotic mixture and maintained in sterile conditions at 37 C in 5% CO_2_ humidified incubator. All the cell lines were tested routinely for the presence of mycoplasma using mycoplasma detection kit (Biotool.Inc, Houston, TX) and found negative.

### Animals

C57Bl/6 mice (6–8 week old, weight 20–25 gm) not more than 6 were kept in individually ventilated cages (IVCs) with standard rodent chow, water ad libitum, and a 12 hour light/dark cycle. The animal experiment protocol (IAEC/166/2012) got prior approval from Institutional Animal Ethics Committee (IAEC) of Rajiv Gandhi Centre for Biotechnology and followed the rules and regulations prescribed by Committee for the Purpose of Control and Supervision of Experiments on Animals (CPCSEA), Government of India. During the course of experiment, the animals were monitored for weight changes and discomfort.

### Azoxymethane (AOM) induced colorectal cancer model

Mice (Male, 6–8 week old) were randomly divided into four groups (n = 10 for all group except group I where n = 5, which serves as the untreated control). Animals in group II-IV received six weekly injections of AOM (10 mg/Kg B.wt, i.p route). Group II was kept as vehicle control; group III started receiving cardamonin (10 mg/kg B.wt, p.o route, 5 days/week) from the first day of AOM injection and continued until the end of the experiment. Group IV started receiving cardamonin 16 weeks after initial AOM injection and continued till sacrifice. All the animals were euthanized at week 30. Colons were excised, and tumors were recorded for each animal. Part of the colon containing tumors was fixed in 10% buffered formalin and remaining portion was snap frozen in liquid nitrogen for RNA and protein extraction.

### Immunohistochemical analysis

The tissue samples were fixed in 10% buffered formalin, dehydrated using different grades of isopropanol and embedded in paraffin. Serial sections of 5 µm were made and used for hematoxylin and eosin staining and Ki-67, staining as described earlier^52^. Briefly, sections were deparaffinised using xylene and rehydrated by passing the slides in different gradient of ethanol and finally to the water. Antigen retrieval was performed using citrate buffer (pH 6) and inactivation of internal peroxidise activity was done using 3%H_2_O_2_ in methanol. Blocking was done using blocking reagent provided by DAKO and the sections were incubated with primary antibody Ki-67(#AbD02531, 1:200) overnight at 4 C and visualized using DAB staining. Hematoxylin was used as counter stain and mounted with DPX.

### Western blotting

Briefly, tissue samples were homogenized in protein lysis buffer and centrifuged at 12000 g for 10 min. The supernatant was collected and used for western blotting. For *in vitro* work, cancer cells were plated in 60mm dishes and treated with the drug for 24hrs. Then lysates were collected in protein lysis buffer and used for further analysis. For nuclear extraction after removing cytoplasmic fraction the pellet was resuspended in nuclear extraction buffer (20 mM Hepes, 400 mM NaCl, 1 mM EDTA, 1 mM EGTA, protease inhibitors). The lysate was vortexed intermittently every 10 mins and repeated for a total period of 1hr on ice. After the stipulated period of incubation, the lysate was centrifuged at 12000 g, 4 °C, 20 minutes. Supernatant was collected and stored at −80 °C till further steps. For SDS-PAGE, 10% Bis acrylamide gels were used and transferred to nitrocellulose membrane. The membrane was blocked with 5% skimmed milk and antibodies; p-JNK(Cell Signaling Technology, #9251, 1:1000), p-p38(Cell Signaling Technology, #9211, 1:1000), p38(Santa Cruz Biotechnology, #sc-535, 1:2000), β-Tubulin(Santa Cruz Biotechnology, #sc-9104, 1:2000), p65(Santacruz, #sc-8008, 1:2000) and β- catenin (Thermo Scientific, #PA5-16429, 1:2000)were added and incubated for overnight at 4^0^ C. HRP conjugated secondary antibody; goat anti rabbit (Jackson laboratories, 1:5000) goat anti-mouse (Santa Cruz Biotechnology, 1:5000) was added and the detection was performed with Biorad ECL solution (BIO-RAD, #1705061).

### Isolation of RNA from tumor samples

The total RNA was isolated using mirVana miRNA isolation kit (Thermofisher Scientific) as per the protocol provided by the manufacturer. Briefly, tumor samples were weighed and grinded with the liquid nitrogen and resuspended in the lysis buffer. Then miRNA homogenate additive and APC was added to the lyaste and centrifuged at 10000 g for 5 min. Then the lysate was passed through filter, washed twice and eluted with water. The yield was determined using a Nanodrop spectrophotometer and integrity of RNA was confirmed by gel electrophoresis.

### MicroRNA profiling of tumor samples

Profiling was performed using GeneChip miRNA Array 4.0 (Affymetrix, Santa Clara, CA) and the procedure was performed based on the manufacturer’s instructions. Briefly, 1000ng of RNA was used for Poly (A) tailing using ATP and PAP enzyme provided by the kit. The tailed RNA was labelled with Biotin for further hybridization. Then it is mixed with Eukaryotic hybridization controls and the entire solution was injected into the array and incubated for 18hrs at 48 °C and 60RPM in the hybridization oven. The Arrays after hybridization were washed and stained with fluidics station 450 and analyzed with GeneChip command console. Microarray data is deposited at GEO with the accession number: GSE93936.

### Analysis of microarray data

Microarray dataset obtained from the four different experimental conditions as provided in the materials and methods was compared as G1 vs G2, G2 vs G3 and G2 vs G4. Raw data obtained as CEL files were normalized using Robust Multi-array Average algorithm^53^. Each Probe ID was mapped to miRBase database (http://www.mirbase.org/). Significant mouse microRNAs were extracted from the list for further functional analysis.

### Validation of selected miRNAs

Independent RNA samples were prepared in parallel with the samples used for the miRNA array as well as from remaining samples in each group. These samples were used to validate the miRNA array data. The validation was done using the quantitative RT-PCR approach which was performed on selected miRNAs targets whose expression was deregulated. Briefly, cDNA was synthesized using 1 µg RNA and RT-PCR was done in a Step One plus (Thermofisher) using specific primers with Taqman assay (Invitrogen). SnoRNA234 and snoRNA202 were served as the internal control. Target gene expression was calculated using 2^−ΔΔCT^ method and expressed as fold change.

### Computational analysis for target genes

To predict the potential targets of the selected miRNAs we used four different target predicting algorithms like miRDB (http://www.mirdb.org/miRDB/) with a cutoff of target score less than 50, TargetScan (http://www.targetscan.org/vert_71/) with a total context score less than −0.36, microT-CDS (http://diana.imis.athena-innovation.gr/DianaTools/index.php?r = microT_CDS/index) with a filter cutoff of 0.8 and miRTarBase (http://mirtarbase.mbc.nctu.edu.tw/). Gene ontology was performed using the Database for Annotation, Visualization and Integrated Discovery (DAVID), Version 6.7 (http://david.abcc.ncifcrf.gov/).

### Validation of selected target genes

The selected target genes of miRNAs were analysed using SYBR green based RT-PCR approach and GAPDH was the internal control. Briefly, the RNA was isolated using trizol method. Then 1.5 µg of it is converted to cDNA using High Capacity cDNA reverse transcription kit. The cDNA was then mixed with gene specific primers (mSLC4A8: F-CGATTGAGTCGTTGTTTGGA, R-AAGGGCATAGTCCTTGCAGA; mRNF20: F-TTGAACTCCTTCCTTGCACA, R-CAATATCCCACTGCAGGTCA; mMEX3 C: F-ACGCCAGGGTTGTAAAATTAAAG, R-GTTTCGAGATGCACGAATCATA; mCYP2C55: F-TCTGTGCTGCATGATGACAA, R-ACGCACATTCGCTTTCCTAT) SYBR green PCR master mix and 20ul was added to each well and run was done in ABI real time instrument. Target gene expression was calculated using 2^−ΔΔCT^ method and expressed as fold change.

### TCGA data analysis for colorectal cancer

TCGA-COAD (Colon adenocarcinoma) data has been downloaded and analyzed using TCGAbiolinks. Read count for selected miRNAs for normal condition and tumor were retrieved and plotted.

### In vitro studies

#### Assay of cell proliferation

The MTT assay was used for accessing cell proliferation as described earlier^52^. Briefly cells were plated in 96 wells (5000 cells/well) and after 24hrs; they were treated with different concentrations of cardamonin for 24, 48 and 72 hours. Wells were washed with PBS and 20 µl of MTT (5 mg/ml) was added to each well, incubated in dark for 4hrs. The formed formazon crystals were dissolved in 100ul of DMSO and readings were taken at 490 nm in TECAN spectrophotometer. Graphs were plotted with optical density on Y-axis.

#### Cell cycle analysis

Cells were plated at a density of 2 × 10^5^ in a 6 well plate and incubated overnight. Next day cells were treated with cardamonin and incubated for 24 and 48hrs. The cells were trypsinized and fixed with 70% alcohol and the RNA degradation was carried out using RNaseA. Then the cells were stained with 10 µl of propidium iodide (PI; 1 mg/ml) for 15 min and analysis was done by flow cytometry.

#### Assay for ROS generation

The reactive oxygen species intensity of the cells was measured using DCFDA staining. Briefly, 2 × 10^5^ cells were plated in 12 welled plates and incubated for 12hrs. The cells were treated with cardamonin, incubated for 24hrs and the media was replaced with 1% FBS containing media with 5 µM H2DCFDA (Thermo Fisher Scientific, #D399), kept for 30 min in dark. The analysis of the cells was done using BD FACS Aria system.

### Assessing loss of mitochondrial membrane potential

To assessed the loss of MMP we used DiOC6(3) (Thermo Fisher Scientific, #D273) stain which accumulates in mitochondria when the membrane is intact. Briefly, 2 × 10^5^ cells were plated in 12 well plates and incubated for 24hrs. Then the cells were treated with cardamonin (20 µM) for 24hrs. Cells were trypsinized and suspended in 0.1 µM DiOC6(3) in serum free media and kept at 37 °C for 15 min in dark. Centrifugation was done at 130 g for 5 min and washed twice with PBS and analysed using flow cytometry.

### Annexin V5-FITC staining for apoptosis

Briefly, cells were plated in 6 well plates at a density of 2 × 10^5^ and incubated overnight. Next day cells were treated with cardamonin for a period of 24hrs. After incubation, they were trypsinised, resuspended in 10X binding buffer and incubated with Annexin V-FITC for 15 min in dark. Then propidium iodide was added and incubated for 5 min and cell populations were analyzed in a flow cytometer.

### Plasmids and generation of stable cells

For generation of stable cell lines the following expression vectors were used. The expression vector pcDNA3 Bax EGFP was provided by Dr. Clark Distelhorst^54^ and vector encoding mitochondrial redox sensing probe (pRA305 Mito-RO-GFP) was provided by Dr. S James Remington^31^. The cells were transfected with lipofectamine LTX (Invitrogen, #15338-100) as per the manufacturer’s protocol. The stably expressing cells were selected by antibiotic selection in 800 µg/ml of Geneticin® (Invitrogen, #11811-031) for four weeks. Further to obtain homogeneously expressing single cell clones, the cells were sorted using BD FACS Aria III cell sorter (BD Biosciences) to enrich cells expressing the fluorescent proteins and homogenously expressing single cell colonies were selected by colony picking method.

### Imaging by microscopy

The assay was done as described earlier^30,55^. The cells homogeneously and stably expressing the probes were seeded in 96 well glass bottom plates (BD Biosciences, #353219) and were incubated at 37 °C for 24 h in a humidified 5% CO_2_ incubator. After 24 h the cells were treated with the cardamonin at appropriate concentrations (in the presence and absence of NAC) prepared in phenol-red free RPMI 1640 (Invitrogen, #11835-030) supplemented with 10% FBS. For imaging of TMRM the respective cells were stained with 100 nM of TMRM (Molecular Probes, #T-668, Eugene, OR, USA) followed by imaging in drug and 20 nM of TMRM containing medium.

Imaging was carried out using a ×40 Plan Apo 0.95 NA objective under an inverted fluorescence microscope (Nikon Eclipse, TE2000-E, Tokyo, Japan). The excitation and emission filter wheels were independently and automatically controlled through NIS element software. The images were acquired using the camera CoolSnapHQ2 (Photometrics, Canada) equipped in microscope. For imaging Bax EGFP, excitation filter of 470 ± 40, emission filter of 525 ± 50 and dichroic 495LP were used. TMRM imaged using a filter combination of Ex: 545 ± 30, Em: 620 ± 60 and dichroic 570LP. Ratio metric imaging for redox sensitive probe Mito RO-GFP was carried out in a dual excitation-single emission mode. The cells were excited using 400 nm and 490 nm sequentially and emission at 515 ± 15 was collected employing a filter turret containing specific dichroic and emission filter.

### Statistical analysis

For microRNA analysis, the comparison of the statistical difference between the groups has been determined through Student’s unpaired t-test using LIMMA package of R-Bioconductor^56^. Significantly expressed miRNAs were identified based on the p-value ≤ 0.05. The RT-PCR values were analyzed using Graph pad Prism 6.0 software.

## Electronic supplementary material

Supplementary table and figure
