# Supplementary material for: Cardamonin inhibits colonic neoplasia through modulation of MicroRNA expression
Source: Sci Rep. 2017 Oct 24;7:13945. doi: 10.1038/s41598-017-14253-8 (PMC5655681; doi:10.1038/s41598-017-14253-8)

Online Supplementary Information

Cardamonin inhibits colonic neoplasia through modulation of MicroRNA expression

Shirley James[1]*, Jayasekharan S. Aparna[1]*, Aswathy Mary Paul[2]*, Manendra Babu Lankadasari [1,3], Sabira Mohammed[1,3], Valsalakumari S. Binu [4], Thankayyan R. Santhoshkumar [1], Girijadevi Reshmi[2], Kuzhuvelil B.Harikumar[1]

Supplementary table 1: Cardamonin inhibited the colorectal cancer formation in mice: Percentage of tumor incidence in each groups expressed as percentage

Group	Number of animals in each group	Tumor incidence	% tumor incidence
1	5	0/5	0
2	8	7/8	87.5
3	10	8/10	80
4	10	7/10	70

Supplementary Table 2: Azoxymethane and cardamonin treatment significantly altered the microRNA profiles in colorectal cancer: Most significant miRNA between groups expressed as fold change

		G1 vs G2	G2 vs G3	G2 vs G4
	miRNA	Fold change	Fold change	Fold change
**a***	mmu-miR-6922-5p	-1.72	1.17	1.16
	mmu-miR-6907-5p	-1.49	1.43	1.13
	mmu-miR-3113-5p	-1.40	1.27	1.60
	mmu-mir-494	-1.05	1.82	1.40
	mmu-mir-3068	-0.83	1.64	1.75
	mmu-miR-690	-0.78	1.43	0.72
	mmu-mir-145a	-0.61	1.16	0.68
	mmu-miR-30b-3p	-0.49	1.17	0.90
**b***	mmu-mir-682	-0.83	0.42	-0.34
	mmu-miR-485-3p	-1.01	-0.86	-0.91
	mmu-miR-299b-5p	-0.93	-0.80	-0.61
	mmu-miR-129-1-3p	-0.63	-0.99	-0.99
	mmu-miR-434-3p	-0.62	-0.92	-0.84
	mmu-mir-485	-0.52	-0.86	-0.91
	mmu-miR-6939-5p	-0.52	-0.61	-0.58
	mmu-miR-6369	-0.47	-0.79	-1.02
	mmu-miR-5100	0.49	-0.67	-0.35

**c***	mmu-miR-7066-3p	0.56	-1.13	-0.61
	mmu-let-7d-3p	1.09	-2.40	-2.01

a*: miRNAs downregulated in AOM alone groups (group 2) and expression levels were increased after cardamonin treatment (group 3 & 4).

b*: There was no statistically significant difference between the groups.

c*: miRNAs upregulated in AOM alone groups (group 2) and expression levels were decreased after cardamonin treatment (group 3 & 4).

Supplementary table 3: Predicted target genes for each miRNA

miRNA	miRDB	TargetScan	microT-CDS	miRTarBase	PolymiRTS
mmu-miR-6922-5p	369	923	209	0	168
mmu-miR-6907-5p	587	1383	418	0	237
mmu-miR-3113-5p	408	3875	176	27	200
mmu-mir-494					
mmu-miR-494-3p	407	355	473	39	265
mmu-miR-494-5p	234	650	55	0	103
mmu-mir-3068					
mmu-miR-3068-3p	407	2366	64	0	123
mmu-miR-3068-5p	169	2395	197	10	134
mmu-miR-690	220	2667	148	27	205
mmu-mir-145a					
mmu-mir-145a-3p	511	4409	388	0	272
mmu-mir-145a-5p	441	621	328	36	179
mmu-miR-30b-3p	252	2649	112	0	166
mmu-mir-682	233	221	193	11	123
mmu-miR-485-3p	100	2421	72	0	86
mmu-miR-299b-5p	99	2155	44	0	89
mmu-miR-129-1-3p	285	414	136	154	153
mmu-miR-434-3p	123	2088	253	37	95
mmu-mir-485					
mmu-miR-485-5p	555	72	161	5	197
mmu-miR-6939-5p	711	3838	433	0	194
mmu-miR-6369	585	530	442	0	194
mmu-miR-5100	13	366	5	1	23
mmu-miR-7066-3p	145	2103	105	0	102
mmu-let-7d-3p	14	312	12	0	35

Supplementary table 4: minimum free energy (MFE) calculated for miRNA-target interaction using the RNAhybrid web service

```
                                                                                        HYBRIDS

CBX2                  5' G     A         UG            AUAU         UG  C 3'            -35.1
                         CC   CUCU    GGUCCC        UCCC     A
                         GG   GAGA    UCAGGG        AGGG    U
mmu-miR-6922-5p       3'       A         UG            G            UG     5'

TNFSF13               5' U        CC          CUU        G             U 3'             -32.8
                        CCUC     UUUGC      UCCC  CUCCCAC
                        GGAG     AGAUG      AGGG GAGGGUG
mmu-miR-6922-5p       3'                      UC                        U 5'

CYP2C55                5'      C       G               U          3'                    -18.8
                             AGG  AGCA  GUC
                             UCC  UCGU  CAG
mmu-let-7d-3p         3' UCUU      G         C      CAUAUC 5'

ZIC1                  5' U                   A      GG      GAAG    A 3'                -26.4
                        GAAGGGCGG  AGG   GUG        AG
                        CUUUCCGUC  UCC    CAU        UC
mmu-let-7d-3p          3' U                 G       AG      A           5'

MEX3C                 5' G      UUGU    U                 C          3'                 -21.3
                        AGA         AG  CAGUGGGUC
                        UCU         UC  GUCGUCCAG
mmu-let-7d-3p         3'         U    C                   CAUAUC 5'

PHEX                  5' G      A                    G              3'                  -25.6
                        CUG  GGAGG GGGCAA
                         GAC CCUCC  UCCGUU
mmu-miR-7066-3p       3'                  G            ACCCAUCU 5'

RNF20                  5' U        CACUG    AA       AAU                G 3'            -30.6
                         UGGG          AG    GGG     GUGGGUAGA
                         ACCC          UC    UCC      UACCCAUCU
mmu-miR-7066-3p       3' G                     CG      GU                    5'

SEC61A2                5'      G          GU                     U 3'                   -32.3
                            CAUCA     ACCAGCUGCCU
                            GUGGU     UGGUCGACGGA
mmu-miR-6939-5p       3' GAAG                                   C 5'

SLC4A8                5'     A            CAG  G                C  3'                   -30.2
                           CCACCA       U  AGCUGCCU
                           GGUGGU       G  UCGACGGA
mmu-miR-6939-5p       3' GAA            U      G                C 5'

SIRT2                 5' A                   AACUCAA                A 3'                -28.7
                         GGGUC CCC             UCCCAGAA
                         CUCGG GGG             AGGGUCUU
mmu-miR-6907-5p       3' G          A      ACAC                      5'

XXYLT1                5' A          CUUA          CCUCUA               C 3'             -31.7
                        GAGCU         UCCCU          UCCCAGAA
                        CUCGG         AGGGA          AGGGUCUU
mmu-miR-6907-5p       3' G                         CAC                    5'
```

Supplementary Table 5: miRNA and the respective targets with their roles in colon cancer.

S.No.	miRNA	Target Gene	Reference
1	mmu-miR-6939-5p	Slc4a8	1
2	mmu-miR-7066-3p	RNF20	2
3	mmu-let-7d-3p	MEX3c	3
4	mmu-let-7d-3p	CYP2C55	4

1. Leviel, Françoise, et al. "The Na+-dependent chloride-bicarbonate exchanger SLC4A8 mediates an electroneutral Na+ reabsorption process in the renal cortical collecting ducts of mice." *The Journal of clinical investigation* 120.5 (2010): 1627-1635.

2. Chernikova, Sophia B., et al. "Deficiency in mammalian histone H2B ubiquitin ligase Bre1 (Rnf20/Rnf40) leads to replication stress and chromosomal instability." *Cancer research* 72.8 (2012): 2111-2119.

3. Burrell, Rebecca A., et al. "Replication stress links structural and numerical cancer chromosomal instability." *Nature* 494.7438 (2013): 492-496.

4. De Robertis, Mariangela, et al. "The AOM/DSS murine model for the study of colon carcinogenesis: From pathways to diagnosis and therapy studies."*Journal of carcinogenesis* 10.1 (2011): 9.

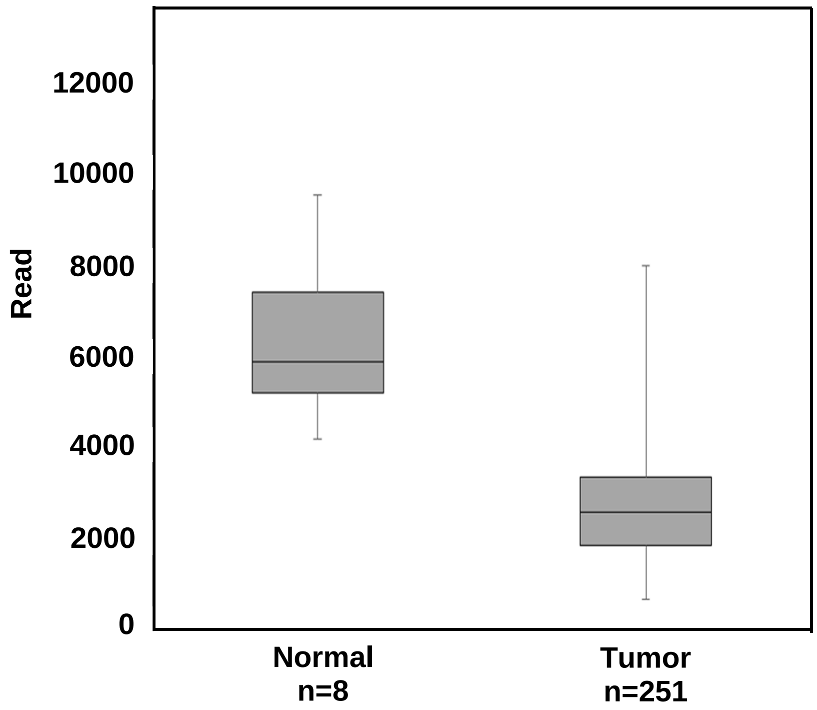

Supplementary figure 1: Expression of hsa-miR-let7d in human CRC samples. Data is expressed as average read count in CRC samples in relative to that of normal samples.

Original blots for figure 1f

Supplementary figure 2

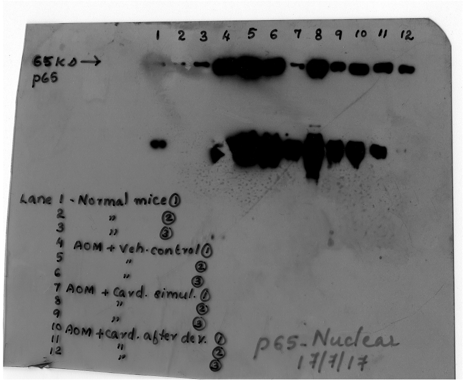

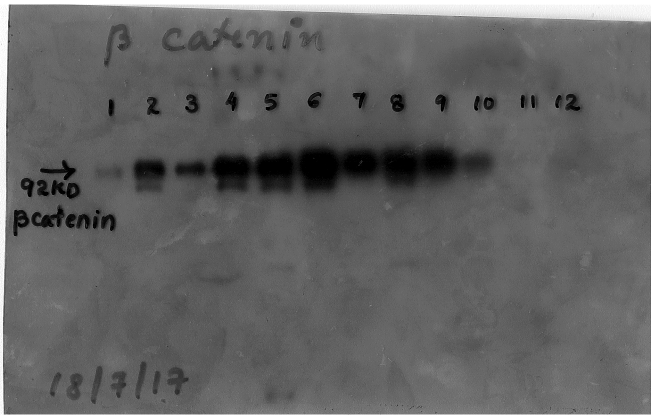

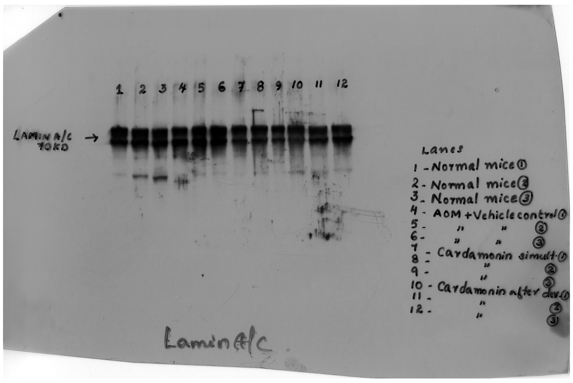

Original blots for figure 8c

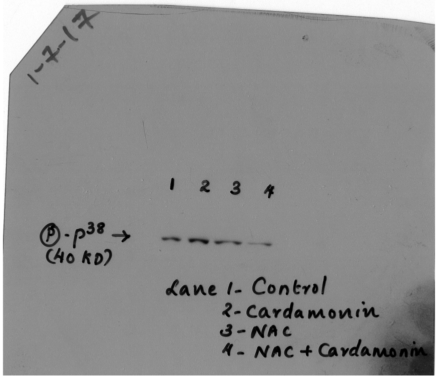

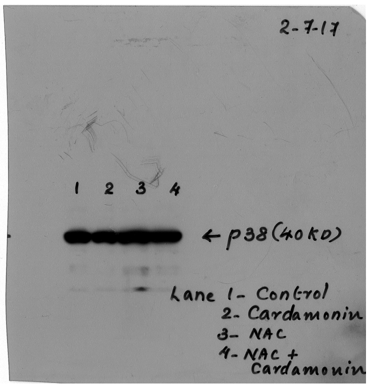

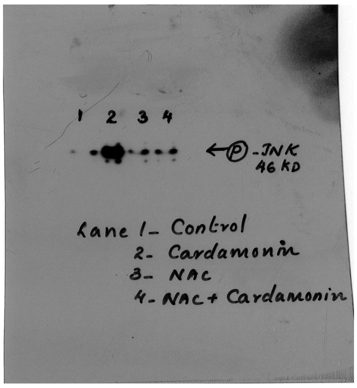

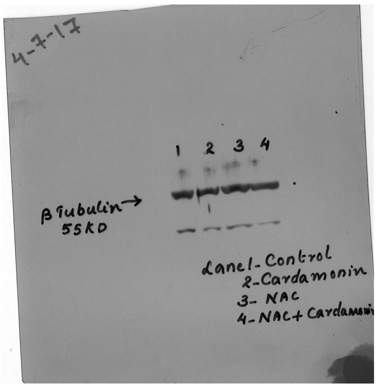

Supplement: Supplementary file 1 — Supplementary table and figure [file 41598_2017_14253_MOESM1_ESM.pdf]
